# Mediators of Inflammation as Targets for Chronic Pain Treatment

**DOI:** 10.1155/2013/783235

**Published:** 2013-11-14

**Authors:** Metoda Lipnik-Stangelj

**Affiliations:** Department of Pharmacology and Experimental Toxicology, Faculty of Medicine, University of Ljubljana, Korytkova 2, 1000 Ljubljana, Slovenia

Treatment of chronic pain remains an unresolved problem in human medicine which greatly impairs quality of life and prolongs treatment. Solving this problem is difficult due to a number of mechanisms and signalling pathways through which chronic pain is generated [1]. A common underlying mechanism of chronic pain is the presence of inflammation at the site of the damaged or affected tissue. The release of proinflammatory and immunoactive substances such as cytokines, neurotrophic factors, and chemokines initiates local actions and can result in a more generalized immune response that leads to the chronic pain condition. Clinical management of chronic pain, that is, neuropathic pain after nerve injury or cancer pain in tumour invasion represents therefore a real challenge due to our limited understanding of the cellular mechanisms that initiate and maintain chronic pain while many of them are closely overlapping with the processes of inflammation, immune response, endocrine and nerve system, and genetic factors as well [2, 3]. This special issue aims to bring a current knowledge of the role of mediators of inflammation in chronic pain, particularly molecular mechanisms, signalling molecules, and their role in the initiation and maintenance of chronic pain, as well as the diagnostic and therapeutic challenges on this field. The brief introductions of nine published papers are as follows.

The involvement of pro- and anti-inflammatory cytokines and angiogenic factors in the pain, associated with brain tumour progression, is shown in the paper entitled “*Cytokine patterns in brain tumour progression*.” R. Albulescu et al. found significant changes in serum levels, with over threefold up-regulation of cytokines IL-6, IL-1*β*, TNF-*α*, and IL-10 and up to twofold up-regulation of cytokines IL-8, IL-2, and GM-CSF, and angiogenic factors VEGF and FGF-2. All these molecules are involved in tumour progression, and are also involved in a generation of pain, associated with disease. While pain is a frequent symptom caused by glioblastoma, the authors concluded that determination of selected cytokines could add to speed and accuracy, making thus possible earlier diagnostics and onset of therapy.

Activation of N-methyl-D-aspartate (NMDA) receptor leads to development of hyperalgesia. In the study called “*posttranslational nitration of tyrosine residues modulates glutamate transmission and contributes to N-methyl-D-aspartate-mediated thermal hyperalgesia*,” C. Muscoli et al., showed that thermal hyperalgesia, induced by intrathecal administration of NMDA, is associated with spinal nitration of GluN1 and GluN2B receptor subunits, glutamine synthase, that normally convert glutamate into nontoxic glutamine, and glutamate transporter. Intrathecal injection of peroxynitrite decomposition catalyst FeTM-4-PyP5+ prevents nitration and inhibits NMDA-mediated thermal hyperalgesia. Their results support the hypothesis that nitration of key proteins involved in the regulation of glutamate transmission is a crucial pathway through which peroxynitrite mediates the development and maintenance of NMDA-mediated thermal hyperalgesia.

Inflammatory conditions, particularly in joint diseases, induce an increase in reactive oxygen substances which have a deleterious role in erosion, osteoarticular degeneration, and pain. L. Di Cesare Mannelli et al. in the psper *“Therapeutic effects of the superoxide dismutase mimetic compound Mn* ^*II*^ *Me*_2_ *DO2A on experimental articular pain in rats”* focused on superoxide dismutases which are decreased in pain conditions like joint inflammation, rheumatoid arthritis, and osteoarthritis. They tested a superoxide dismutase mimetic compound 4,10-dimethyl-1,4,7,10 tetraazacyclododecane-1,7-diacetic acid Mn^II^ complex (Mn^II^Me_2_DO2A), which has potently relieved a pain in arthritis models, and they showed that the effect differed from a direct inhibition of cyclooxygenase enzymes. In chronic administration, it involved prevention of tissue degenerative alterations induced by the oxidative stress and reduction of a persistent inflammatory pain via a direct antioxidant mechanism, while in acute administration, it may decrease the nociceptive nervous fiber activation induced by the local production of reactive oxygen substances. Given these properties and the low toxicity of the molecule, Mn^II^Me_2_DO2A represents a novel compound potentially suitable for the treatment of inflammatory and neuropathic pain.

In the paper “*Neurovascular unit in chronic pain*,” B. M. Radu et al. focused on the role of blood-brain barrier (BBB) and blood-spinal cord barrier (BSCB) during the development of chronic pain. Reviewing several inflammatory- and nerve-injury-based pain models, they argue that the clarification of molecular BBB/BSCB permeabilization events is necessary for understanding chronic pain mechanisms. They proposed that the understanding of chronic pain mechanisms would benefit from the extension of research efforts to the neurovascular unit as a whole and reviewed the available evidence on the interaction between analgesic drugs and the neurovascular unit. Furthermore, they discussed chronic pain comorbidities, such as neuroinflammatory and neurodegenerative diseases, in a view of neurovascular unit changes, and innovative pharmacological solutions, targeting neurovascular unit components in chronic pain treatment. 

Pain perception displays large interindividual variability in the population that affects selection of analgesics and their dosing. In the comprehensive paper “*Pharmacogenetics of chronic pain and its treatment*,” S. Světlík et al. reviewed the most recognized pharmacogenetic areas and variables in the treatment of chronic pain. They focused on the impact of genetic variability of drug metabolizing enzymes, transporters, receptors, and pathways involved in chronic pain perception and on the efficacy and safety of analgesics and other drugs used for chronic pain treatment. Although several candidate genes have been identified in the literature, there is only limited clinical evidence substantiating for the penetration of the testing for these candidate biomarkers into the clinical practice. While the pain-perception regulation and modulation are still not fully understood, the authors have concluded that more complex knowledge of genetic and epigenetic background for analgesia will be needed prior to the clinical use of the candidate genetic biomarkers.

In autoimmune diseases of the nervous system, the neuropathic pain is frequently presented. In the review article “*Neuropathic pain in animal models of nervous system autoimmune diseases*,” D. H. Tian et al. focused on neuropathic pain, associated with multiple sclerosis and Guillain-Barre syndrome, as well as with experimental autoimmune encephalomyelitis and experimental autoimmune neuritis, in animal models which enable investigations of behavioural changes, underlying mechanisms, and potential pharmacotherapeutic approaches for neuropathic pain, associated with these diseases. In this review, the symptoms, mechanisms, and clinical therapeutic options in these conditions are examined, and the value of experimental autoimmune encephalomyelitis and experimental autoimmune neuritis animal models for the study of neuropathic pain in multiple sclerosis and Guillain-Barre syndrome is highlighted.

In the paper “*Chronic pain treatment: the influence of tricyclic antidepressants on serotonin release and uptake in mast cells*,” I. Ferjan M. Lipnik-Štangeli discussed the role of serotonin (5-HT), tricyclic antidepressants, and mast cells in the generation of chronic pain in the periphery and central nerve system. They showed that, besides inhibition of the pain stimuli in the central nerve system, 5-HT might be associated also by an increased pain transmission from the periphery, where mast cells play an important role. The authors demonstrated that tricyclic antidepressants are able to influence mast cell-derived 5-HT levels via at least three different mechanisms: secretion of 5-HT, uptake of exogenous 5-HT, and reuptake of secreted 5-HT. They concluded that analgesic effect of tricyclic antidepressants involved different mechanisms of action.

Current evidence indicates lines of the prominent role of gonadal hormones in affecting pain occurrence and intensity. In the review article “*Testosterone-induced effects on lipids and inflammation*,” S. Vodo et al. described interesting aspects on the generation of chronic pain, influenced by androgen hormones, particularly testosterone, and lipids, whose altered metabolism is often accompanied by the release of interleukins and lipid-derived pro-inflammatory mediators, and based on interactions which are often not considered in chronic pain mechanisms. Also important is the ability of pain as well as pain therapies to affect gonadal hormone metabolism. The authors concluded that lower testosterone levels are associated with an increased metabolic risk, systemic inflammation, and chronic pain.

In the paper “*Inflammatory pain and corticosterone response in infant rats: effect of 5-HT1A agonist buspirone prior to gestational stress*,” hypothalamo-pituitary-adrenal axis and serotonin system interactions in the chronic pain are discussed. I. P. Butkevich et al. presented the effect of buspirone on the dynamics of the inflammatory pain-like behaviour and stress response of corticosterone during the formalin test in the infant male rat offspring and evaluated the correlation between pain-like and hormonal parameters. They concluded that maternal buspirone, applicated before the stress during gestation, may enhance an adaptive mechanism of the inflammatory nociceptive system through activation of the hypothalamo-pituitary-adrenal axis peripheral link.
